# Spatial immune profiling of bone-metastatic castration-resistant prostate cancer reveals radium-223 immunomodulates the bone-tumor microenvironment

**DOI:** 10.1038/s41391-025-01069-1

**Published:** 2025-12-26

**Authors:** Paul G. Corn, Guoyu Yu, Yibo Fan, Miao Zhang, Patricia Troncoso, Devaki Shilpa Surasi, Suyu Liu, Jaffer A. Ajani, Christopher J. Logothetis, Guocan Wang, Theocharis Panaretakis, Sue-Hwa Lin

**Affiliations:** 1 Departments of Genitourinary Medical Oncology, The University of Texas MD Anderson Cancer Center, 77030 Houston, TX USA; 2 Departments of Translational Molecular Pathology, The University of Texas MD Anderson Cancer Center, 77030 Houston, TX USA; 3 Departments of Gastrointestinal Medical Oncology, The University of Texas MD Anderson Cancer Center, 77030 Houston, TX USA; 4 Departments of Pathology, The University of Texas MD Anderson Cancer Center, 77030 Houston, TX USA; 5 Departments of Nuclear Medicine, The University of Texas MD Anderson Cancer Center, 77030 Houston, TX USA; 6 Departments of Biostatistics, The University of Texas MD Anderson Cancer Center, 77030 Houston, TX USA; 7 MD Anderson Cancer Center UTHealth Graduate School of Biomedical Sciences, 77030 Houston, TX USA

**Keywords:** Prostate cancer, Translational research

## Abstract

**Background:**

Prostate cancer (PCa) bone lesions are bone-forming, which contribute to an immunosuppressive bone-tumor microenvironment (bone-TME). Targeting PCa-induced bone with the radiopharmaceutical Radium-223 (Ra223) is approved for men with bone-metastatic castration-resistant PCa (bmCRPC). We studied the effect of Ra223 on spatial localization of tumor and immune cells in bmCRPC tumor biopsies to gain insights into therapeutic immunomodulation of the bone-TME.

**Methods:**

Transiliac bone marrow biopsies were performed at baseline and end of treatment (EOT) in 24 patients with bmCRPC treated with Ra223. In three patients with paired specimens containing tumor, multiplex immunofluorescence was performed with antibodies for a tumor cell marker (CK), T-cell markers (CD3, CD4, CD8, FOXP3, and granzyme B), macrophage markers (CD68 [total macrophages] and CD206 [M2-like macrophages]).

**Results:**

In all three patients, we observed enrichment of CD68^+^ macrophages, around tumor-induced bone at baseline. CD3 staining revealed T cell depletion where tumor cells formed large sheets but not where tumor cells were scattered. The effects of Ra223 on tumor cells and T cell subsets were heterogeneous. In one patient, tumor cells were reduced, and CD3^+^/CD8^+^ T cells were increased. In a second patient who evidenced osteolytic progression, tumor cells were increased while CD3^+^/CD8^+^ T cells were decreased. In a third patient, there was high number of tumor cells at baseline that modestly decreased and the number of CD3^+^/CD8^+^ T cells modestly increased after Ra223 treatment.

**Conclusions:**

The changes in tumor cell numbers after Ra223 treatment seemed to be inversely correlated with changes in CD3^+^/CD8^+^ and CD4^+^/FoxP3^+^ cells, but not with other immune cells, in these 3 patients. Further characterization of immune cells in the bone-TME will be required before developing strategies to enhance immunotherapy efficacy in bmCRPC.

**Clinical trial registration:**

This study does not report the clinical results of a clinical trial, but it does use samples from a completed clinical trial that is registered with clinicaltrials.gov (NCT02135484).

## Introduction

While localized prostate cancer (PCa) is frequently curable, bone-metastatic PCa (bmPCa) is not. Whereas bone metastases in most solid tumors are lytic, bmPCa are predominantly osteogenic, a phenotype that explains the high sensitivity of Technetium-99 methylene diphosphonate (99mTc-MDP) bone scans in detecting lesions for diagnosis and staging [1]. Lytic lesions are generally not detected on 99mTc-MDP bone scans but are visible on conventional CT scans. While PET-imaging using 18F-fluciclovine, F-18 based PMSA, or FDG tracers may increase the sensitivity of detecting lytic lesions compared to conventional CT scans, osteoblastic lesions predominate [2, 3]. Osteogenic PCa-induced bone formation promotes tumor progression and confers therapy resistance [4–9]. We recently demonstrated that PCa-induced bone originates from endothelial-to-osteoblast (EC-to-OSB) transition stimulated by tumor-secreted BMP4 [10]. BMP4 activates multiple pathways that coordinately reprogram ECs into osteoblasts [11]. EC-OSB hybrid cells secrete tenascin C, which promotes PCa metastasis through YAP/TAZ inhibition [9]. Together, these studies indicate that PCa cells reprogram the bone-TME to promote tumor progression.

The approval of radium-223 (Ra223) for bone metastatic castrate-resistant PCa (bmCRPC) validated the bone-TME as a therapy target. Ra223 is a high-energy α-emitting calcium mimetic that localizes to newly formed matrix within osteogenic metastases and induces double-strand DNA breaks in adjacent cells. In the randomized phase 3 placebo-controlled ALSYMPCA clinical trial, targeting PCa-induced bone with Ra223 improved the median overall survival in men with bmCRPC. Interestingly, Ra223 treatment produced only modest reductions in prostate-specific antigen (PSA), and there was no correlation between reductions in PSA level and clinical outcomes. In contrast, Ra223 treatment produced substantial reductions in alkaline phosphatase, a marker for osteoblasts, and reductions were associated with improved clinical outcomes [12, 13]. These data suggest that the clinical efficacy of Ra223 is mediated primarily through targeting osteoblasts rather than PCa epithelial cells in the bone-TME. However, the biological effects of Ra223 on the bone-TME remain unclear.

BmPCa is relatively unresponsive to immune checkpoint therapy [14]. We recently found that PCa-induced EC-OSB hybrid cells secreted paracrine factors, including Wnts, CXCL14, and LOX, which induced M2 macrophage polarization and recruited M2-like macrophages to the tumor-induced bone area, leading to an immunosuppressive bone-TME [15]. We also found that Ra223 treatment of SCID mice bearing osteogenic PCa xenograft tumors decreased F4/80^+^ macrophages (mouse macrophages) and CD206^+^ macrophages (M2-like macrophages) in tumors [15]. These observations suggest that Ra223 treatment can potentially immunomodulate the bone-TME.

In this study, we examined the effect of Ra223 on spatial localization of tumor and immune cells in bmCRPC tumor biopsies to gain insights into therapeutic immunomodulation of the bone-TME. We employed multiplex immunofluorescence (mIF) to permit spatial analysis of multiple cell types in the bone-TME. Due to interpatient heterogeneity of bone metastases related to intrinsic tumor properties and prior treatments [16, 17], we performed pairwise comparisons of mIF using each patient’s baseline and end of treatment (EOT) samples as internal controls. In addition, mIF results were contextualized with 99mTc-MDP bone scans and 18F-NaF-PET/CT for each patient.

## Materials and methods

### Patients

We previously conducted a clinical trial of Ra223 in patients with bmCRPC [18]. From October 9^th^, 2014 to September 29^th^ 2015, twenty-five patients received Ra223 dichloride 50 kBq (0.0014 mCi)/kg intravenously every 4 weeks for up to six doses. Technetium-99 methylene diphosphonate (99mTc-MDP) bone scans and ^18^F-sodium fluoride positron emission tomography/computed tomography (18F-NaF-PET/CT) scans were performed at baseline and EOT. 99mTc-MDP bone scans are standard for evaluation of bone metastases, and 18F-NaF-PET/CT scans were investigated given their potential for superior image quality, higher sensitivity, and better spatial resolution in the detection of bone metastases [19, 20]. Trans-iliac bone marrow biopsies were performed at baseline and EOT (week 24). Of 25 patients who enrolled and were treated, 24 had biopsies performed at baseline, and eight had tumor in the biopsy specimen. Of these eight patients, three also had tumor in the EOT biopsy specimen. Paired baseline and EOT bone specimens from these three patients (1, 9, and 20) were then further analyzed.

### Bone biopsy specimens

Trans-iliac bone marrow biopsies, including both aspirate and cores, were collected. The aspirate is collected using a 15 g Illinois needle. The core is collected using a11g Jamshidi needle. Bone marrow cores decalcification is as follows. Bone marrow core is fixed in 10% neutral buffered formalin for 12–16 h. Adequate fixation of the bone marrow tissue in 10% buffered formalin is crucial before decalcification to preserve cellular structures. The samples were then decalcified in a combination of formic acid/formalin solution (Fisher Chemical – Cal-Ex II - CAT#: CS511-4D) for 4 h. The decalcified samples were washed in running distilled water for 10 min and samples were placed in 70% ethanol until ready to load into processor for making paraffin blocks.

### Multiplex immunofluorescence

Baseline and EOT biopsy specimens from patients 1, 9, and 20 were fixed in formalin and decalcified in formic acid before being embedded in paraffin. Bone sections (5 µm) on slides were laid flat and incubated overnight in a 60°C oven. Bone sections were subjected to immunostaining with an optimized eight-plex panel of antibodies for T-cell markers (CD3, CD4, and CD8), T-cell function markers (FoxP3 and granzyme B), macrophage markers (CD68 and CD206), and a PCa epithelial marker (CK), then hematoxylin-eosin (H&E) staining was performed. mIF with the eight markers was performed by Ultivue technology (Cambridge, MA, USA). Number of cells of 12 fields from each sample were presented.

### Image analysis

The QuPath cell detection program was used in comparing the relative mean fluorescence intensity between baseline and EOT samples of each marker. Area of 345,006 um^2^ was selected as a field. For each sample, a total of 12 fields were selected for analysis. The number of cells positive with two specific markers from the 12 selected fields were determined by using RStudio. Thresholds were set for each marker, and the same threshold was used across all 6 samples. Of note, mean fluorescence intensity analysis using QuPath cannot distinguish markers that also express in different cell types. For example, CD206 antibody may stain endothelial cells besides macrophages. The total cell counts for cells that were positive for two markers increase the specificity of assigned cell type, however, the analysis may be biased due to the threshold applied for each marker.

### Statistical analysis

For pairwise comparisons of mean fluorescence intensity of 12 fields, Student’s t-test was used. *P* < 0.05 was considered statistically significant.

## Results

### Clinical information of patients

Clinical information on the three patients (1, 9 and 20) with paired baseline and EOT bone-tumor biopsies containing tumor is shown in Table 1. Per CHAARTED imaging criteria [21], patients 1 and 20 had “high-volume” disease and patient 9 had “low-volume” disease. Patients 1 and 20 also had elevated baseline levels of total alkaline phosphatase (TAP). Patients 1 and 9 completed all six doses of Ra223. While patient 20 experienced an initial decline in PSA after the first two doses, there was a rapid increased in PSA, total alkaline phosphatase and cancer-associated pain consistent with disease progression after the third dose and treatment was discontinued.

**Table 1:** Clinical information of patients.

Patient no.	Total no. of Ra223 doses	Baseline TAP level, U/L^*^ (normal 38-126 U/L)	EOT TAP level, U/L^*^	% Change in TAP	Overall survival, months	Baseline PSA level, ng/mL	EOT PSA leve, ng/mL	% Change in PSA level	Prior treatments
1	6	268	59	-77%	33.8	8.4	0	-100%	Leuprolide, bicalutamide
9	6	94	49	-49%	31.5	301.3	672.2	+123%	Leuprolide, abiraterone, docetaxel plus caboplatin, selinexor
20	3	681	484	-29%	5.3	4170	1886	-54.7%	Leuprolide, enzalutamide, sipuleucel-T, cabazitaxel, abiraterone

*TAP, total alkaline phosphatase activity.

### CD68^+^CD206^+^ M2-like macrophages were enriched in the bone-TME at baseline

To examine the suitability of the bone biopsy specimens for immunostaining, we performed immunohistochemistry (IHC) for expression of M2-like macrophage marker CD206 on formalin-fixed, decalcified, paraffin-embedded tissue from the baseline sample of patient 20. We found abundant CD206^+^ staining in areas around tumor-induced bone (Supplementary Fig. 1), consistent with our previous finding that M2-like macrophages are enriched in tumor-induced bone areas [15]. Following confirmation of IHC suitability, to evaluate the numbers and spatial localization of macrophages and T cells in the bone-TME, baseline and EOT biopsy specimens from patients 1, 9, and 20 were immunostained with an optimized eight-plex panel of antibodies for T-cell markers (CD3, CD4, and CD8), T-cell function markers (FoxP3 and granzyme B), macrophage markers (CD68 and CD206), and a PCa eptihelial marker (CK), and then performed hematoxylin-eosin (H&E) staining.

**Fig. 1:**
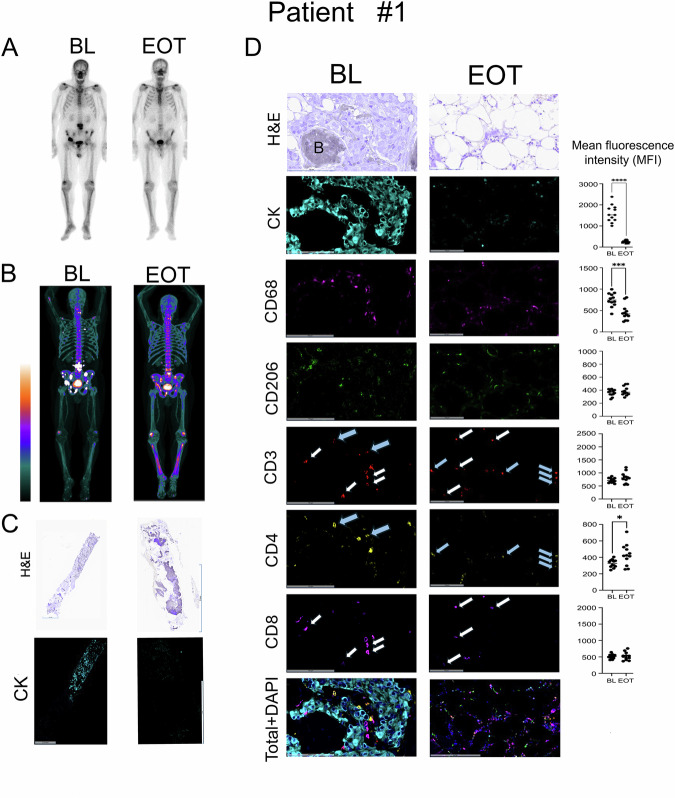
Characteristics of patient 1 at baseline and end of treatment. **A** 99mTc-MDP Bone scans before and after Ra223 treatment, anterior views. Between baseline and EOT, there was marked reduction in radiotracer uptake within multifocal skeletal metastases and resolution of some foci. BL, baseline. EOT, end of treatment. **B** 18F-NaF-PET/CT bone scans before and after Ra223 treatment show a significant positive response to Ra223 therapy. Color scale: Blue/green indicates background uptake, and orange/yellow indicates sites of disease. **C** H&E staining and CK immunostaining of bone biopsy specimens. A significant amount of woven bone was found in the bone marrow. CK staining showed that the majority of marrow space was occupied by PCa cells in baseline bone biopsy specimens. The level of PCa cells is decreased in EOT bone biopsy. **D** Tumor and immune cell staining. CK staining showed a marked reduction in the amount of tumor cells after Ra223 treatment. Mean fluorescence intensity (MFI) of individual immune cell types in the bone-TME before and after Ra223 treatment were shown in the right panels. White arrowheads: CD3^+^/CD8^+^ cells. Blue arrows: CD3^+^/CD4^+^ cells. B, bone. *,*p* <0.05, **,*p* <0.01, ***,*p* <0.001, ****,*p* <0.0001.

In patient 1, the baseline 99mTc-MDP bone scan showed uptake in the lumbar spine, acetabula, bilateral iliac bones, right ischium, left femoral head, and posterior aspect of the left fifth rib (Fig. 1A). 18F-NaF-PET/CT confirmed multifocal osseous metastases and as predicted, revealed more lesions than the 99mTc-MDP bone scan (Fig. 1B). The TAP level was elevated (Table 1), consistent with aberrantly active PCa-induced bone formation. H&E staining showed a significant amount of woven bone (Fig. 1C). PCa cells, identified by CK staining, occupied the majority of the marrow space (Fig. 1C), and tumor cells clustered and formed sheets surrounded by woven bone (Fig. 1D, Supplementary Fig. 2). CD68^+^ cells, representing total macrophages, were the most abundant immune cells. CD206 was used as an M2-like macrophage marker. Immune cells that expressed both CD68 and CD206 markers were counted as M2-like macrophages. We found that a significant portion of CD68^+^ cells were also positive for CD206 (Fig. 1D, Supplementary Fig. 2), suggesting enrichment of M2-like macrophages. Few CD8^+^ cells were present in the tumor-induced bone area. No CD8^+^ cells were detected within the tumor clusters, but CD68^+^ and CD206^+^ cells were detected within the tumor clusters (Supplementary Fig. 2A). These observations suggest that T cells, but not macrophages, were excluded from the tumors. The relative numbers of tumor and immune cells are shown in Supplementary Fig. 2B.

**Fig. 2:**
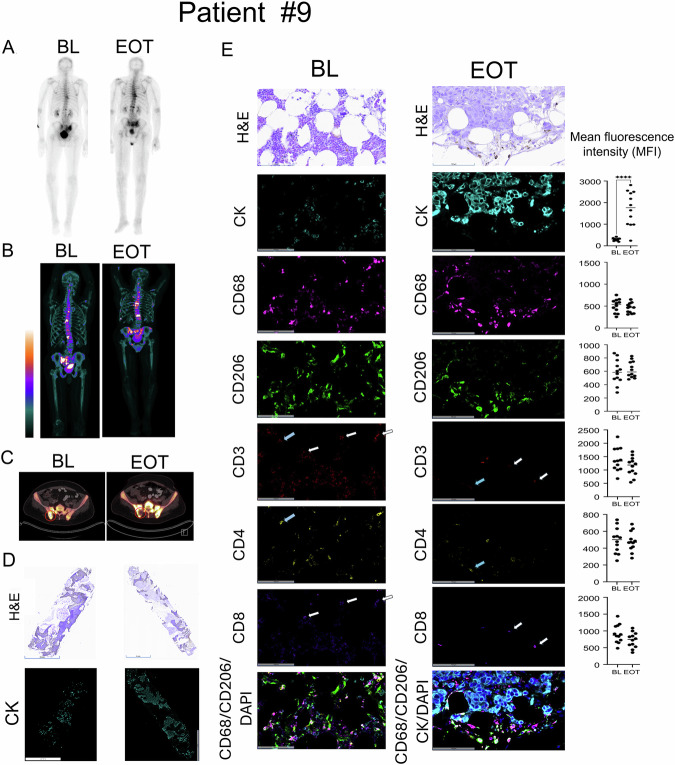
Characteristics of patient 9 at baseline and end of treatment. **A** 99mTc-MDP Bone scans before and after Ra223 treatment, posterior views. Between baseline and EOT, there were mixed changes, with some areas of improvement and some new lesions indicative of progression. BL, baseline. EOT, end of treatment. **B** 18F-NaF-PET/CT bone scans before and after Ra223 treatment show progression of osseous metastases. Color scale: Blue/green indicates background uptake, and orange/yellow indicates sites of disease. **C** Several lesions became progressively osteolytic and destructive, such as along the ventral aspect of the low sacrum on the right (red circle). **D** H&E staining and CK immunostaining of bone biopsy specimens. A significant amount of woven bone was found in the bone marrow. CK staining showed that the level of PCa cells is low in baseline bone biopsy specimen. In EOT bone biopsy specimen, the majority of marrow space was occupied by PCa cells. **E** Tumor and immune cell staining. CK staining showed an increase in the level of tumor cells after Ra223 treatment. In EOT bone biopsy specimen, PCa cells were abundant and formed large sheets of tumor cells. Mean fluorescence intensity (MFI) of individual immune cell types in the bone-TME before and after Ra223 treatment were shown at the right panels. White arrowheads: CD3^+^/CD8^+^ cells. Blue arrows: CD3^+^/CD4^+^ cells. B, bone. ****, *p* < 0.0001.

In patient 9, the baseline 99mTc-MDP bone scan showed uptake at L1, the left posterior iliac bone, the sacrum, and left sacroiliac joint (Fig. 2A). 18F-NaF-PET/CT confirmed and revealed more metastases than the 99mTc-MDP bone scan (Fig. 2B). The TAP level was in the normal range (Table 1), suggesting less active disease. H&E staining showed woven bone (Fig. 2D). There were fewer metastatic PCa cells in the baseline specimen compared with patient 1, and the cells were dispersed throughout the normal-looking bone marrow in a scattered pattern (Fig. 2E, Supplementary Fig. 3A). CD68^+^ and CD206^+^ cells were abundant and localized along the bone surface as well as intermixed with the scattered PCa cells (Fig. 2E, Supplementary Fig. 3A). Relative baseline numbers of CD3^+^ and CD8^+^ cells in patient 9 were higher compared with patient 1 (Supplementary Fig. 2B vs. 3B).

**Fig. 3:**
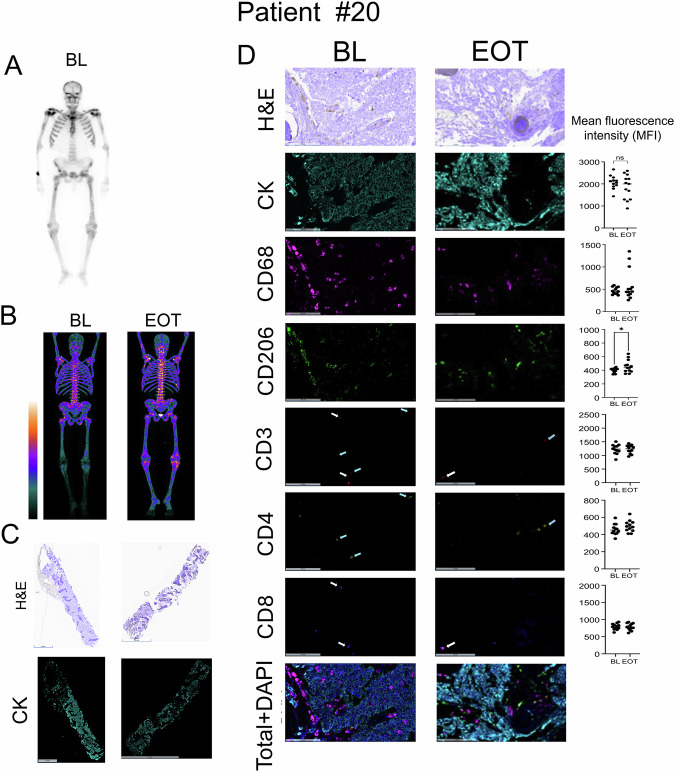
Characteristics of patient 20 at baseline and end of treatment. **A** The baseline 99mTc-MDP bone scan of Patient #20, anterior view. Extensive diffuse abnormal osseous uptake in the skull, spine, bilateral ribs, bilateral pelvic bones, bilateral femur, bilateral humeri and the sternum. **B** 18F-NaF-PET/CT bone scans before and after Ra223 treatment show an increase in the extent of osseous metastases and a subtle increase in sclerosis in the distal femurs and proximal tibias. Color scale: Blue/green indicates background uptake, and orange/yellow indicates sites of disease. BL, baseline. EOT, end of treatment. **C** H&E staining and CK immunostaining of bone biopsy specimens. **D** Tumor and immune cell staining. CK staining showed a high number of tumor cells that clustered as sheets and almost completely filled the marrow space. Mean fluorescence intensity (MFI) of individual immune cell types in the bone-TME before and after Ra223 treatment were shown at the right panels. White arrowheads: CD3^+^/CD8^+^ cells. Blue arrows: CD3^+^/CD4^+^ cells.

In patient 20, the baseline 99mTc-MDP bone scan showed multifocal disease in the skull, spine, left and right ribs, pelvic bones, femurs, humeri, and sternum (Fig. 3A). 18F-NaF-PET/CT confirmed osseous metastases throughout the axial and appendicular skeleton (Fig. 3B). The TAP level was highly elevated, consistent with increased bone activity, and patient 20 had the highest volume of disease by imaging (Fig. 3A). H&E staining showed a high amount of woven bone (Fig. 3C) and CK^+^ tumor cells almost completely filled the space as sheets of tumor cells surrounded by woven bone (Fig. 3D, Supplementary Fig. 4A). CD68^+^ and CD206^+^ macrophages were present within the tumor sheets and in the interface between bone and tumor cells (Fig. 3D, Supplementary Fig. 4A). Very few CD3^+^, CD4^+^, or CD8^+^ cells were detected **(**Fig. 3D, Supplementary Fig. 4A). The numbers of immune cells are shown in Supplementary Fig. 4B.

The numbers of tumor cells and immune cells in the three baseline bone biopsies are shown in Supplementary Fig. 4C and D to facilitate comparison. The specimens differed in the amount of tumor cells and tumor cell distribution patterns, consistent with previous reports that bmCRPC is a heterogeneous disease [16, 17]. All three patients had a high proportion of CD68^+^ total macrophages. The numbers of CD3^+^/CD8^+^ cells were very low in patients 1 and 20, both also had high numbers of tumor cells. In patient 9 specimen that had a relatively low level of tumor cells, CD3^+^/CD8^+^ cells were abundant and distributed among scattered tumor cells. In contrast, in the patient 20 specimen that had a relatively high level of tumor cells, CD3^+^/CD8^+^ cells were sparce and excluded from the sheets of tumor cells.

### Effect of Ra223 on immune cells in the bone-TME

**Fig. 4:**
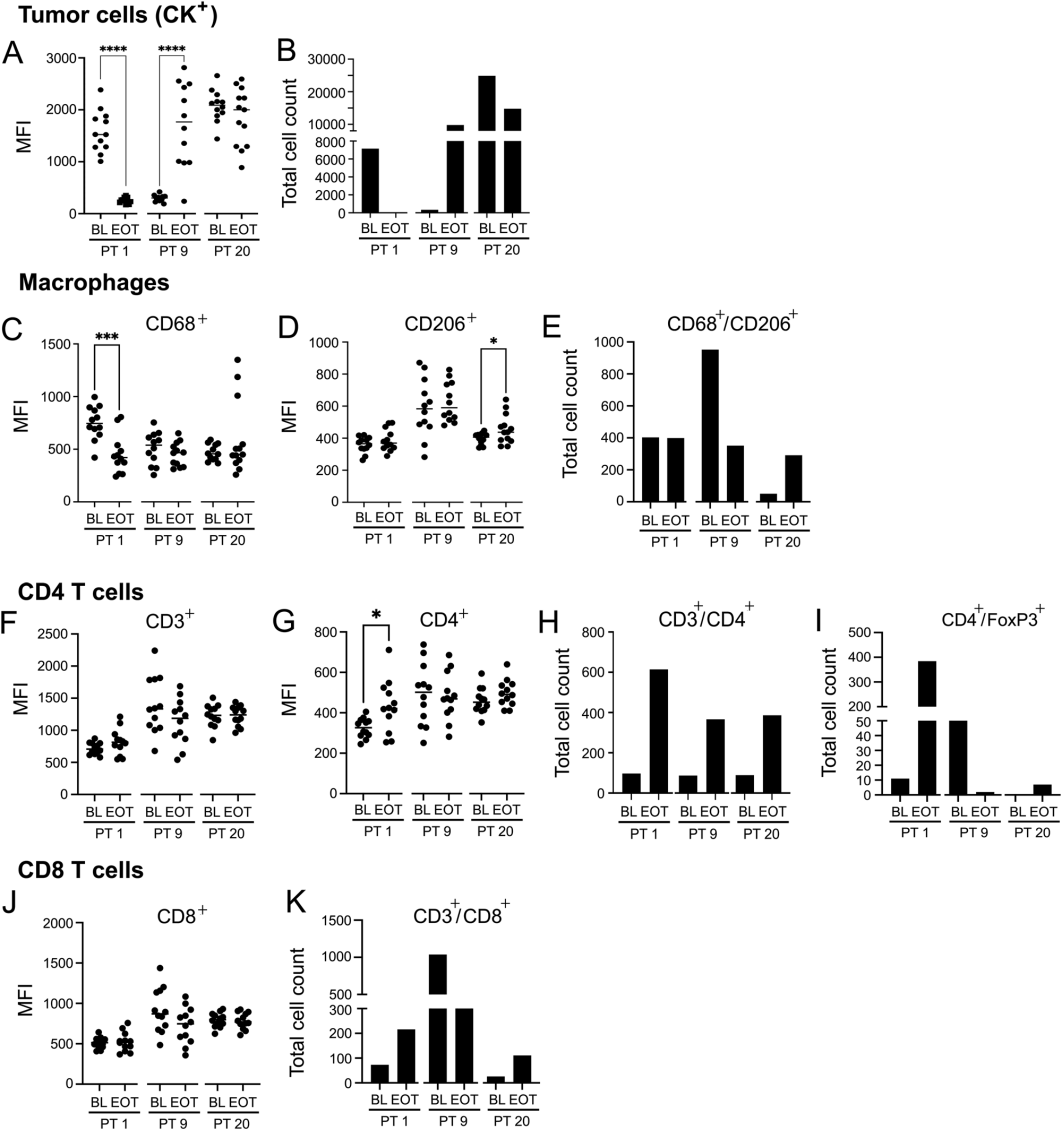
Relative mean fluorescence intensity and cell numbers of different immune cell types at baseline and end of treatment of patient 1, 9, and 20. **A** Mean fluorescence intensity (MFI) of tumor cells. 12 fields from each bone biopsy were analyzed. **B** Total tumor cell counts from the 12 fields. **C** MFI of CD68 cells. **D** MFI of CD206 cells. **E** Total counts of cells positive for both CD68 and CD206. **F** MFI of CD3 cells. **G** MFI of CD4 cells. **H** Total counts of cells positive for CD3 and CD4. **I** Total counts of cells positive for both CD4 and FoxP3. **J** MFI of CD8 cells. **K** Total counts of cells positive for both CD3 and CD8. The tumor cells were significantly reduced in patient 1 and significantly increased in patient 9 at end-of-treatment (EOT) compared to baseline (BL). There was a significant decrease in the MFI of CD68^+^ and increase in the MFI of CD4^+^ cells after Ra223 treatment in patient 1. The MFI of CD206^+^ cells of Patient 20 was significantly increased after Ra223 treatment. *,*p* < 0.05, **,*p* < 0.01, ***,*p* < 0.001, ****,*p* < 0.0001.

To determine the effects of targeting PCa-induced bone with Ra223, we next compared the EOT results on scans and immunostaining with the baseline results. In patient 1, the 99mTc-MDP bone scan showed marked reduction in radiotracer uptake and resolution of some foci (Fig. 1A). 18F-NaF-PET/CT also showed a significant positive response to therapy (Fig. 1B). There was a 77% decrease in the serum TAP level (from 268 to 59 U/L) (Table 1). Immunostaining showed a marked reduction in the number of tumor (CK^+^) cells (Fig. 1C, 1D). Whereas PCa cells at baseline appeared as sheets, PCa cells at EOT appeared dispersed in the bone marrow in an scattered pattern (Fig. 1D). The quantification of tumor cells and immune cell subtypes at baseline and EOT for the patients 1, 9, and 20 are summarized in Fig. 4. For patient 1, there was a trend towards a decrease in the numbers of CD68^+^ macrophages (Fig. 1D, 4C), and the numbers of CD3^+^/CD4^+^ and CD3^+^/CD8^+^ cells were increased (Fig. 4H, 4K).

In patient 9, the 99mTc-MDP bone scan showed mixed changes, with some areas of improvement and some new lesions (Fig. 2A). 18F-NaF-PET/CT showed progression of osseous metastases, with the skeletal lesions becoming osteolytic and destructive (Fig. 2B, C). The serum TAP level decreased after Ra223 treatment (Table 1). There was a significant increase in the number of tumor cells in the biopsy specimen (Fig. 2D, E). Furthermore, whereas PCa cells at baseline showed a scattered pattern, PCa cells at EOT were abundant and formed sheets (Fig. 2E). These observations suggested disease progression despite Ra223 treatment. The numbers of CD68^+^/CD206^+^ macrophages and CD3^+^/CD8^+^ cells were decreased (Fig. 4E, K).

Patient 20 did not have an EOT 99mTc-MDP bone scan but an EOT 18F-NaF-PET/CT showed an increase in the extent of osseous metastases (Fig. 3B). Despite the lack of clinical treatment benefit, there was a reduction in the TAP level, although the level remained much higher than normal (Table 1). At EOT, tumor cells almost completely filled the marrow space, similar to what was observed in the baseline biopsy specimen (Fig. 3C, D). CD3^+^/CD8^+^ cells were modestly increased (Fig. 4K).

The mean fluorescence intensity from 12 selected fields of tumor cells and immune cell subsets at baseline and at end-of-treatment for the 3 paired biopsies is summarized in Fig. 4. We also performed a total count of cells double positive for markers (i.e. CD68^+^/CD206^+^, CD3^+^/CD4^+^, CD4^+^/FoxP3^+^, CD3^+^/CD8^+^) within the 12 fields (Fig. 4 E, H, I, K). We found that the changes in tumor cell numbers after Ra223 treatment seemed to be inversely correlated with changes in CD3^+^/CD8^+^ cells (Fig. 4B, K), but not with other immune cells, in these 3 patients.

Because we have included granzyme B, a marker for activate CD8 T cells, and FoxP3, a marker for regulatory T-cells, in our multiplex immunofluorescence staining, we analyzed the CD8^+^/Granzyme B^+^ subtype of T-cells in the 3 paired sets of bone biopsies. Because most of CD8^+^ cells were found to be also CD3^+^, we determined the number of CD8^+^/Granzyme B^+^ cells in the samples. We found that in the baseline samples, CD8^+^/Granzyme B^+^ subtype accounts for 5% (6/111, 11 fields), 0% (0/170 CD8, 5 fields), and 0% (0/63 CD8^+^, 11 fields) of total CD8^+^ cells in patient 1, 9, and 20 samples, respectively (Supplementary Figs. 5–7). In the Ra223-treated samples, CD8^+^/Granzyme B^+^ subtype accounts for 7% (4/54, 11 fields), 2% (2/89, 15 fields), and 4% (1/24, 8 fields) of total CD8^+^ cells in patients 1, 9, and 20 samples, respectively. We also quantified the CD4^+^/FoxP3^+^ subtype of T-cells in the 6 bone biopsies (Fig. 4I). The changes in CD4^+^/FoxP3^+^ cell numbers seem to be inversely correlated with changes in tumor cells in these 3 patients.

## Discussion

In this study, we used mIF to compare the effects of Ra223 on the spatial localization of tumor and immune cells in bmCPRC. We observed two general patterns of tumor cell distribution within the bone-TME: (i) sheets of tumor cells occupying marrow space between woven bone, and (ii) scattered tumor cells dispersed within otherwise normal-appearing bone marrow. Enrichment of macrophages, especially M2-like macrophages, was seen in both tumor patterns. In contrast, T cells were excluded from tumor cells in a sheet pattern, but not in tumor cells in a scattered pattern. The changes in tumor cell numbers after Ra223 treatment seemed to be inversely correlated with changes in CD3^+^/CD8^+^ and CD4^+^/FoxP3^+^ cells, but not with other immune cells, in these 3 patients. Further characterization of immune cells in the bone-TME will be required before developing strategies to enhance immunotherapy efficacy in bmCRPC.

T-cell exclusion in TMEs has been described in many cancer types [22]. Interestingly, in the baseline bone biopsy specimen of patient 9, who had relatively low number of total CK^+^ tumor cells, T cells were dispersed within scattered tumor cells. In contrast, in the baseline bone biopsy specimens of patients 1 and 20, both of whom had relatively high number of total CK^+^ tumor cells, T cells were “excluded” from large clusters of tumor cells. It has been shown that in TMEs, T cells are depleted by tumor cells [23], macrophages [24], and other cells across a spectrum of different tumor types [25]. Our data support a model whereby distinct tumor cell architectures may influence the spatial distribution of immune cells with large tumor cell clusters inversely correlating with the presence of T cells in the bone-TME.

We previously showed that M2-like macrophages present in osteogenic tumors in mice inhibit CD8^+^ T-cell proliferation and cytotoxic activity [15]. Genetic or pharmacological inhibition of PCa-induced EC-to-OSB transition reduced M2-like macrophages [15]. However, we only detected a decrease in CD68^+^ macrophages and a trend towards an increase in the number of CD3^+^/CD8^+^ cells in patient 1 after treating with Ra223. It is possible that the extent of macrophages reduction by Ra223 used in clinical setting may not be sufficient to reverse CD8^+^ T-cell paucity in patients. It may also be possible that additional mechanisms besides those mediated by PCa-induced bone are involved in CD8^+^ T-cell paucity in the bone-TME. Ra223 effects on CD3^+^ /CD4^+^ and CD4^+^/FoxP3^+^ T cells in the bone-TME were similarly heterogeneous. Our sample of 3 patients was biased towards those with accessible tissue. Given variable baseline disease characteristics and response to treatment, these patients may not represent the general characteristics of a broader population of bmCRPC patients receiving Ra223. Thus, additional studies of more patients will be required to further characterize the mechanisms of immunosuppression in the bone-TME before developing strategies to enhance immunotherapy efficacy in bmCRPC.

Our studies illustrate the technical challenges of studying the bone-TME in patients with bmCRPC. We and others have found that transiliac bone biopsies in patients with visible pelvic metastases on imaging produce tumor-containing samples in only approximately 35% to 45% of patients, and the incidence of serially positive samples is lower [26, 27]. At the time this trial was conducted from October 9^th^, 2014, to September 29^th^, 2015. Our yield of 32% baseline positive biopsies in this study was consistent with our earlier efforts. While CT image-guided biopsies increase the rate of positive biopsies, the cores are generally smaller than with transiliac bone marrow biopsies which can reduce the yield of tissue for analysis [28]. However, were we to design the trial today, we certainly would have considered interventional radiology (IR)-directed approaches. The development of “liquid biopsies” in the form of transcriptome profiling of extracellular vesicles, which provides information on tumor, immune and stromal cells in the bone-TME [18], may improve longitudinal monitoring of bmPCa.

We have previously characterized immune cell distribution using osteoblastic metastases generating under subcutaneous model [15]. We found that M2-like macrophages are recruited to the area of tumor-induced bone, and the M2-like macrophages were able to suppress the cytotoxic activity of CD8^+^ T-cells in vitro. It is of interest to note that in the bone marrow with infiltrating tumor cells of bmPCa bone biopsies, CD68^+^/CD206^+^ M2-like macrophages were enriched around tumor-induced bone, and CD8^+^/Gzm B^+^ T cells were significantly reduced, similar to those observed in the subcutaneous model. However, whether the distribution of other immune cell subtypes is also similar requires further study.

This study has important limitations. The sample size was small due to the technical challenges described above, and the number of immune markers analyzed was limited. We did not examine the effect of Ra223 on natural killer cells, which were reported to be reduced in bmCRPC [29]. The bone biopsy specimens obtained from individual patients before and after Ra223 treatment might have been obtained from different tumor deposits depending on placement of the biopsy needle. Furthermore, because we used a trans-iliac bone marrow biopsy approach, we only acquired bone biopsies for study from patients with pelvic bone disease. However, bone biopsies performed on the iliac crest may not be representative of the other parts of the skeleton due to a relatively higher content of trabecular bone [30, 31]. Thus, our results are not generalizable because the immune bone-TME in a pelvic bone might be different than in another bones (e.g. spine).

### Final paragraph

Our results show that bone-TME of bmCRPC is enriched with M2-like macrophages and a paucity of T cells, and Ra223-mediated targeting of tumor-induced osteoblasts can lead to changes in bone-TME. These findings may inform the development of rational combination strategies that target PCa-induced bone to enhance immunotherapy approaches in bmCRPC.

## Supplementary information

Supplemental Material

## Data Availability

Data is available to any reader upon request. Please email the corresponding author to request the data.
