# Supplementary material for: Spatial immune profiling of bone-metastatic castration-resistant prostate cancer reveals radium-223 immunomodulates the bone-tumor microenvironment
Source: Prostate Cancer Prostatic Dis. 2025 Dec 26;29(3):602–10. doi: 10.1038/s41391-025-01069-1 (PMC13472971; doi:10.1038/s41391-025-01069-1)
Supplement: Supplementary file 1 [file 41391_2025_1069_MOESM1_ESM.docx]

**Supplementary Figures**

**Spatial immune profiling of bone-metastatic castration-resistant prostate cancer reveals radium-223 immunomodulates the bone-tumor microenvironment**

**Supplementary Figure 1. Immunohistochemistry for the expression of CD206 in a baseline sample of patient 20.** CD206^+^ staining was found in areas around tumor-induced bone. T, tumor. B, bone.

**Supplementary Figure 2. Characteristics of patient 1 baseline bone biopsy.**

(A) Tumor and immune cell staining. Tumor cells (CK^+^) clustered and formed sheets of tumor cells surrounded by woven bone. Macrophages (CD68^+^) were abundant, and the majority were also CD206^+^. Very few CD4^+^ and CD8^+^ cells were present (white arrowheads). Some CD4^+^ cells were also FoxP3^+^ (white arrowheads). (B) Numbers of immune cells from 12 fields.

**Supplementary Figure 3. Characteristics of patient 9 bone biopsy at baseline.** (A) Tumor and immune cell staining. The number of CK^+^ PCa cells were low, and these cells appeared scattered in the normal-looking bone marrow. CD68^+^ cells and CD206^+^ macrophages were abundant and localized along the bone surface as well as intermixed with the dispersed PCa cells. CD8^+^ cells were abundant and could be easily detected (white arrowheads). CD3^+^, CD4^+^, and CD8^+^ cells were commingled with scattered tumor cells. Very few CD4^+^/FoxP3^+^ cells were detected. (B) Numbers of immune cells from 12 fields.

**Supplementary Figure 4. Characteristics of patient 20 bone biopsy at baseline.** (A) Tumor and immune cell staining. CD68^+^ and CD206^+^ macrophages were present within the tumor sheets and in the interface between bone and tumor cells. Very few CD3^+^, CD4^+^, and CD8^+^ cells were detected. (B) Numbers of immune cells from 12 fields. (C) Numbers of tumor cells from 12 fields in patients 1, 9, and 20 baseline bone biopsies. The number of tumor cells differed. (D) Numbers of immune cells from 12 fields in patients 1, 9, and 20. All three patients had a high proportion of CD68^+^ total macrophages. CD3^+^/CD8^+^ cells were very low in patients 1 and 20, both also had high numbers of tumor cells.

**Supplementary Figure 5. CD8/Gzm B subtype of patient 1 bone biopsy at baseline.** Very low CD8^+^ and granzyme B (Gzm B)^+^ cells were present. Three fields with 8, 8, and 10 CD8^+^ cells for field #1, #2, and #3, respectivley, were shown. One CD8^+^/Gzm B^+^ cells (while arrow) in field #1 was found while no CD8^+^/Gzm B^+^ cells were found in field #2, or #3. Of the 111 CD8^+^ cells in a total of 11 fields, six of them (0.05%) are Gzm B^+^. B, bone.

**Supplementary Figure 6. CD8/Gzm B subtype of patient 9 bone biopsy at baseline.** Three fields with 27, 13, and ~50 CD8^+^ cells for field #1, #2, and #3, respectivley, were shown. While CD8^+^ cells are relatively abundant, no CD8^+^/Gzm B^+^ cells were found. B, bone.

**Supplementary Figure 7. CD8/Gzm B subtype of patient 20 bone biopsy at baseline.** Very low CD8^+^ and granzyme B (Gzm B)^+^ cells were present. Three fields with 3, 10, and 17 CD8^+^ cells was found for field #1, #2, and #3, respectivley. No CD8^+^/Gzm B^+^ cells was found. One CD8^-^/Gzm B^+^ cell (Green arrow) was found in field #3. B, bone.
